# The Use of Acceleration to Code for Animal Behaviours; A Case Study in Free-Ranging Eurasian Beavers *Castor fiber*

**DOI:** 10.1371/journal.pone.0136751

**Published:** 2015-08-28

**Authors:** Patricia M. Graf, Rory P. Wilson, Lama Qasem, Klaus Hackländer, Frank Rosell

**Affiliations:** 1 Faculty of Arts and Sciences, Department of Environmental Sciences, Telemark University College, Bø i Telemark, Norway; 2 Department of Integrative Biology and Biodiversity Research, Institute of Wildlife Biology and Game Management, University of Natural Resources and Life Sciences, Vienna, Vienna, Austria; 3 Swansea Moving Animal Research Team, Biosciences, College of Science, Swansea University, Singleton Park, Swansea, Wales, United Kingdom; CNRS (National Center for Scientific Research), FRANCE

## Abstract

Recent technological innovations have led to the development of miniature, accelerometer-containing electronic loggers which can be attached to free-living animals. Accelerometers provide information on both body posture and dynamism which can be used as descriptors to define behaviour. We deployed tri-axial accelerometer loggers on 12 free-ranging Eurasian beavers *Castor fiber* in the county of Telemark, Norway, and on four captive beavers (two Eurasian beavers and two North American beavers *C*. *canadensis*) to corroborate acceleration signals with observed behaviours. By using random forests for classifying behavioural patterns of beavers from accelerometry data, we were able to distinguish seven behaviours; standing, walking, swimming, feeding, grooming, diving and sleeping. We show how to apply the use of acceleration to determine behaviour, and emphasise the ease with which this non-invasive method can be implemented. Furthermore, we discuss the strengths and weaknesses of this, and the implementation of accelerometry on animals, illustrating limitations, suggestions and solutions. Ultimately, this approach may also serve as a template facilitating studies on other animals with similar locomotor modes and deliver new insights into hitherto unknown aspects of behavioural ecology.

## Introduction

Understanding animal behaviour is fundamental to biology, and an important precondition for other fields of research [1]. Indeed, the behaviours that animals adopt have profound consequences for lifetime reproductive success [2, 3], reason enough to study them and their consequences. Ethologists have used different approaches to document animal behaviour, of which direct observation and radio-tracking are probably the most common [4]. However, these methods have limitations, particularly with regard to shy or elusive species, or those that live in inaccessible habitats. Recently, scientists have recognized the potential of accelerometers in animal-attached loggers to identify animal behaviour [5–7]. Acceleration forces produce a signal that depends on the orientation of the device (and when fixed to a subject, therefore, the posture of that subject), often called the ‘static’ acceleration, onto which is superimposed a ‘dynamic’ signal derived from the subject motion [8].

Initial applications using animal-borne accelerometers have mainly focused on marine species, where researchers had none or only limited access to investigate their study species’ behaviour in their natural habitats [9]. A vast majority of these studies were conducted on birds including penguins [5, 10, 11], other seabirds such as cormorants [12, 13], boobies and gannets [14, 15], but also marine mammals such as seals [16, 17] and cold-blooded animals such as turtles [18, 19] and a range of fish species [20–22]. In land-based studies, accelerometers have been used on domestic animals [23, 24], livestock [25, 26] and other animal species in captivity [27, 28]. Moreover, there is a range of studies on terrestrial birds both in captivity and in the wild including many raptor species [29–31]. The field is rapidly growing and in the last years, accelerometers have also been increasingly used on free-living terrestrial mammalian species [32–34].

Specific applications vary from investigations of body movements to elucidate foraging behaviour [35, 36], particularly as relates to diving behaviour [5, 37], to travel speed [38, 39], to flight dynamics [30, 40, 41], to animal welfare issues [42, 43], to construction of activity-budgets [44, 45] and even time/energy activity budgets [46–48]. The latter can be derived from both overall- or vectorial dynamic body acceleration (ODBA or VeDBA, respectively). Both ODBA and VeDBA produce, calibrated by means of one of the common methods for determining energy expenditure in animals [49, 50], a powerful predictor of movement-based energy expenditure [12, 51].

In this study, we deployed tri-axial acceleration loggers on free-ranging and captive beavers *Castor spp*. to examine how acceleration coded for behaviour. Beavers are large, territorial rodents that feature a monogamous mating system [52]. They live in family groups and defend their territories aggressively against intruders [53]. As strict herbivores, they feed on tree bark and other herbaceous plants including aquatic plants [54]. Beavers are semi-aquatic and combine four-legged locomotion on land with aquatic locomotion (swimming, diving) in water. Moreover, beavers are nocturnal and exhibit a hidden lifestyle that is difficult to trace with conventional methods (see above). Due to this and their complex behavioural repertoire, beavers form a perfect study system for our approach.

To reinforce and complete the field of accelerometry studies, we present a framework for identifying a suite of behaviours in beavers. By using this approach, we aim to shed light onto several aspects of beaver behaviour and present an innovative, non-invasive method that can be used to remotely quatifiy the behaviour of free-living animals. We suggest that our study should provide researchers with a starting point for constructing behaviour-, time-, or even energy budgets and thus provide an important cornerstone to understanding animal behavioural ecology.

## Materials and Methods

### Free-ranging animals

The study was carried out between April-November 2009–2011, and September-October 2011 on a population of free-ranging Eurasian beavers *C*. *fiber* in south-eastern Norway (59°23’ N, 09°09’ E, Telemark). The study sites were the three rivers Gvarv, Saua and Straumen, which flow through a semi-agricultural and mixed woodland landscape. Beavers have inhabited the study area since the 1920s [55]. Since 1997, the animals have been part of a live-trapping program with an extensive capture-mark-recapture scheme [56]. Captured animals are routinely sexed [57], weighted, micro-chipped (ID-100A Microtransponder, Trovan Electronic Identification Devices LTD., Hull, UK) and marked with unique colour-plastic (Ovine Rototags or Research Minitags, Dalton Continental BV, Lichtenvoorde, The Netherlands) or metal (1005–3 Self piercing monel ear tag, National Band and Tag Co., Kentucky, USA) ear-tag combinations [56].

### Live-trapping and attachment of acceleration loggers

We caught 12 known adult Eurasian beavers (8 males—mean mass 21.7 ± 1.5 kg and 4 females–mean mass 23.1 ± 2.6 kg) from 10 different territories. Beavers were located during the night (usually from 9:00 pm – 6:00 am) using a hand-held spotlight, approached by boat, identified by their ear-tags and caught by jumping into the water with a landing net, following the method established by Rosell and Hovde [58]. The animals were then transported onto the shore, and transferred into a cloth bag where handling and tagging procedures were undertaken without the use of anaesthesia. Tags, consisting of an eight-channel acceleration logger (JUV Elektronik, Schleswig-Hollstein, Germany, size 90 x 15 mm dia—mass 62 g) and a VHF-transmitter (Reptile Glue-On R1910, Advanced Telemetry Systems, Minnesota, USA, size 35 x 18 x 8 mm—mass 10 g), were attached on the fur of the lower back using two-component epoxy resin. Before attachment, the tags were connected with wire and integrated in 4.5 mm half-mesh net covering (Mørenot Fishery AS, Møre og Romsdal, Norway). The whole unit was 130 x 90 mm in size (incl. netting) and weighed 90 g in air, which accounts for 0.69% of the body weight of the lightest beaver (weighing 13 kg, see below) used in this study. We attached all units identically at precisely 15 cm above the tail along the spine because device orientation was critical for this study. Overall handling time was no more than 20 minutes and beavers were released at the trapping site where they swam calmly away. Pictures were taken of all animals to ascertain tag orientation. After one to three weeks the animals were recaught and the units removed from the fur, using a scalpel to cut the guard hair, leaving the underfur unaffected.

### Tri-axial acceleration loggers

The tri-axial acceleration loggers used in this study included orthogonally placed sensors for tri-axial acceleration (-4 to +4 *g*) and pressure transducers (range 950 to 10,000 mB). The three acceleration channels were so placed within the logger and the logger so placed on the animal, that they represented the animal’s dorso-ventral axis (heave), the anterior-posterior axis (surge) and the lateral axis (sway) [7]. Data (22 bit resolution) were recorded at 8 Hz and 2 Hz for acceleration and pressure, respectively, and stored on a 1 GB memory card. The loggers were programed using a simple serial port (COM) terminal emulation program (Terminal by Bray pp).

### Control animals

We conducted control observations to determine how accelerometer signal related to behaviour in three zoos. Tri-axial acceleration loggers were deployed on; (i) two North American beavers *C*. *canadensis* (one male, mass 22 kg and one female mass 19 kg) in the Alpenzoo Innsbruck, Austria during June 2009, (ii) a male Eurasian beaver (mass 13 kg) in the Highland Wildlife Park in Kincraig, United Kingdom and (iii) on a female Eurasian beaver (mass 19 kg) in Edinburgh Zoo, United Kingdom, both in February 2010. In September 2011, we also observed two device-equipped free-ranging Eurasian beavers (one male, one female) in our study area. We observed and/or filmed both captive and free-living animals with a hand-held video camera (NVGS-180, Panasonic Corporation, Osaka, Japan) for three to four hours a day over a week. The clock times on the video-camera and the acceleration logger were synchronised to allow us to compare the recorded beaver behaviours with the acceleration data. In captivity, we observed the animals from outside their enclosure and used a small head-mounted torch during dark periods. Elevated paths, observer platforms and glazed windows guaranteed good visibility of the focal animals. All enclosures contained at least one water basin that enabled the animals to swim and was deep enough to dive. One of the basins in the Alpenzoo had a ground-level glazed front, which allowed for detailed observations of body postures during diving. In addition, the two animals from Alpenzoo had a glazed window into their sleeping chamber so we were able to observe them as they slept or moved within this space. Rocks, soil, grass and concrete stretches were available in all enclosures and promoted land-based activities such as standing, walking, feeding and grooming. Furthermore, the animals were provided with branches and twigs of woody plants, vegetables and fruit to stimulate feeding behaviour on different food items. In the wild, all observations were conducted from a boat, using binoculars and a spotlight when needed. Data collected during observation periods were excluded from analysis and no other tagged beavers were present in the respective territories. Neither the presence of the observer nor the use of light had any detectable influence on the subject (see e.g. [59]).

### Data analysis

Downloaded data were inspected using custom-made software (Snoop, Swansea University, UK) and compared to video tapes from zoos and notes from direct observations to identify behaviours and associated data sequences. Analyses on free-ranging animals were started on day two after capture to avoid possible effects of the handling process. Data from both sexes and different seasons was pooled since beavers are sexually monomorphic and we did not intend to compare time budgets but rather focused on the implementation of physical movements. We exported 10 examples for each behaviour, using 10 seconds of walking and standing (more transient behaviours) and one minute of swimming, grooming and feeding behaviours (behaviours which occur over long periods). Sleeping consists of a whole sequence of lying postures, thus sleeping sessions were inspected visually only and ODBA values (see below) were recorded. After ensuring that our transducers were properly calibrated, we derived the static acceleration signal from each axial sensor by using a running mean over 2 seconds [7]. In this study, the overall dynamic body acceleration (ODBA) was used to relate to the animals’ movement and activity [7, 60], respectively. ODBA was calculated by subtracting the derived static acceleration from each acceleration channel (see above) and calculating the absolute product from consequent dynamic acceleration values for each behaviour (see [12]).

Statistical analyses were conducted in R 2.14.1 (R Foundation for Statistical Computing, Austria) and OriginPro 8.5.1 (OriginLab Corporation, USA). Accelerometry data have been analysed by a range of machine learning algorithms [31, 61] and we used random forests (R package randomForest, n = 500 trees, mtry = 2, Liaw & Wiener 2002) to classify our data. Random forests are a powerful statistical classifier for acceleration data [31] that include variable importance measures for each predictor variable [62, 63]. All behaviours, apart from sleeping (see above for rationale), were categorized using mean values and standard deviations of surge, sway, heave acceleration and ODBA.

### Ethics statement

The study, including all handling and tagging procedures (for details see above), was approved by the Norwegian Experimental Animal Board (id 742, id 2170) and the Norwegian Directorate for Nature Management (2008/14367 ART-VI-ID), which also granted permission to conduct fieldwork in our study area. Subsequent observations of individuals with acceleration loggers indicated no obvious differences in behavioural patterns of tagged and untagged individuals. The patch of clipped guard hair grew back within three to four months.

## Results

**Fig 1:**
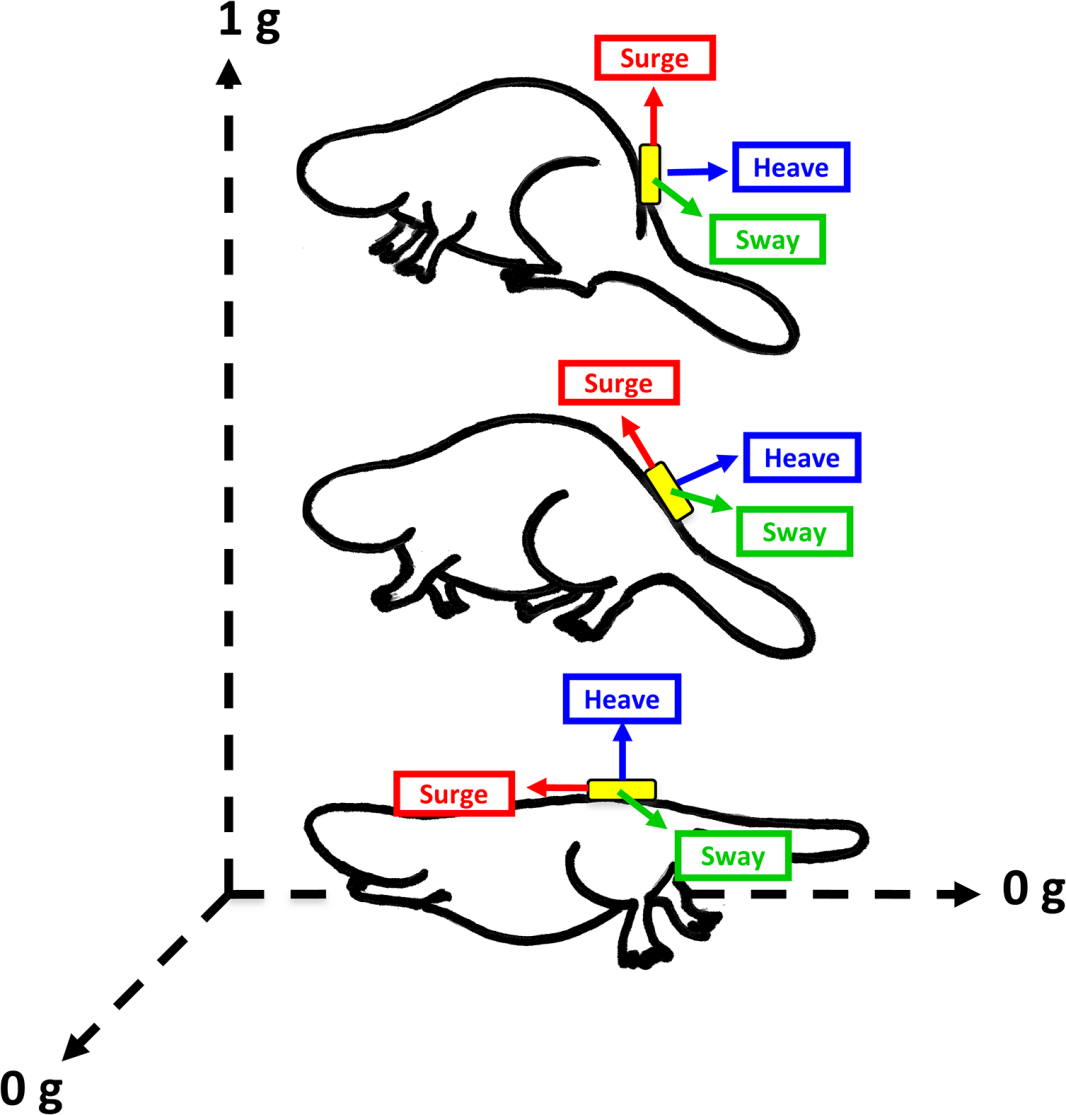
Changes in Eurasian beaver posture. Changes in orientation of the surge, sway and heave acceleration axes are illustrated during standing, walking and swimming (top to bottom).

**Fig 2:**
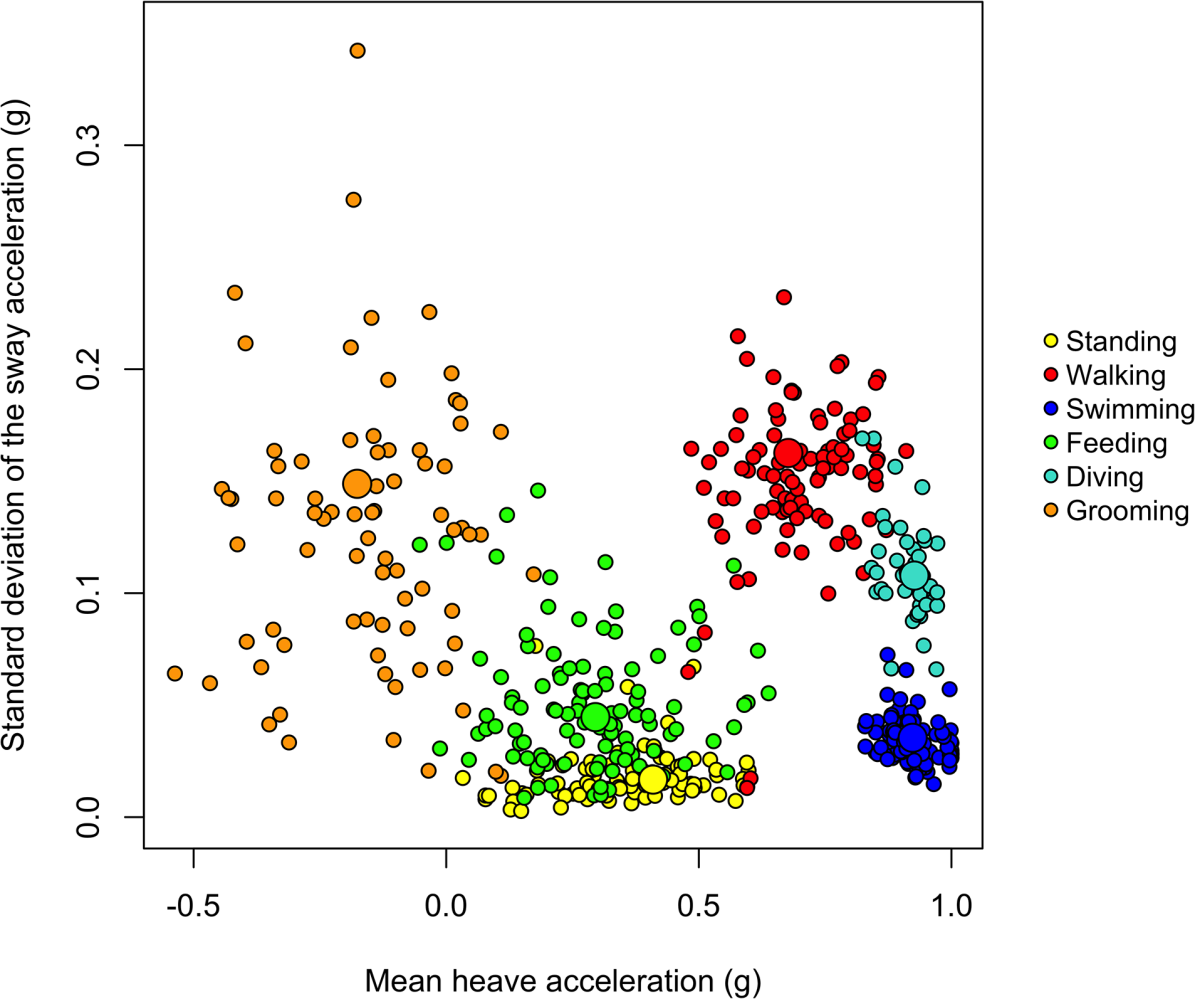
Class center plot for Eurasian beaver behaviours. The plot contains prototypes (big dots) for the six behavioural classes modelled by random forests. Prototypes are median values of samples with the largest number of k-nearest neighbours of the same class. In this way, they mark the center of each class and indicate how the variables relate to the classification.

Overall, we identified seven different behaviours including standing, walking, swimming, feeding, grooming, diving and sleeping. All behaviours could be clearly differentiated by the static acceleration signal from the beavers during captive studies, and all of which were recorded by the free-living animals. During individual observations, we captured at least 10 video sequences for each of the seven behaviours, lasting for a minimum of 10 seconds. The unit’s positioning on the lower back facilitated, in particular, distinguishing postures in water (flat, stretched out) from postures on shore, as illustrated for standing, walking and swimming in Fig 1. Our random forests model classified behaviours with 95% accuracy (see Table 1 for all goodness-of-fit metrics). Kappa statistics were over 0.94 and the model showed high specificity (100%) and sensitivity values (95.8%), indicating substantial predictions with low cross-classification error. All data points clustered out according to their class affiliation, with only minor overlap between standing and feeding (with cross-tabulated class errors of 0.08% and 0.10%, respectively; Fig 2). The class center for feeding (the prototype) indicated that feeding involves animals leaning more backwards than with simply standing (with mean heave values closer to 0) because the front part of the body is more upright during processing of food items (Fig 2). The greater spreading of grooming values along the y-axis in Fig 2 shows the variability of this behaviour, in particular with regard to lateral movement. This is largely owing to variable time periods spent on grooming either the right or the left side of the body (Fig 2).

**Table 1:** Accuracy measures for the random forests model predicting class affiliation to six different Eurasian beaver behaviours.

Metric		Value (%)
	Out-of-bag error rate	4.84
Cross-validated	Accuracy	94.99
	Specificity	100.00
	Sensitivity	95.79
	Error rate	5.01
	Cohen’s Kappa	93.90

**Fig 3:**
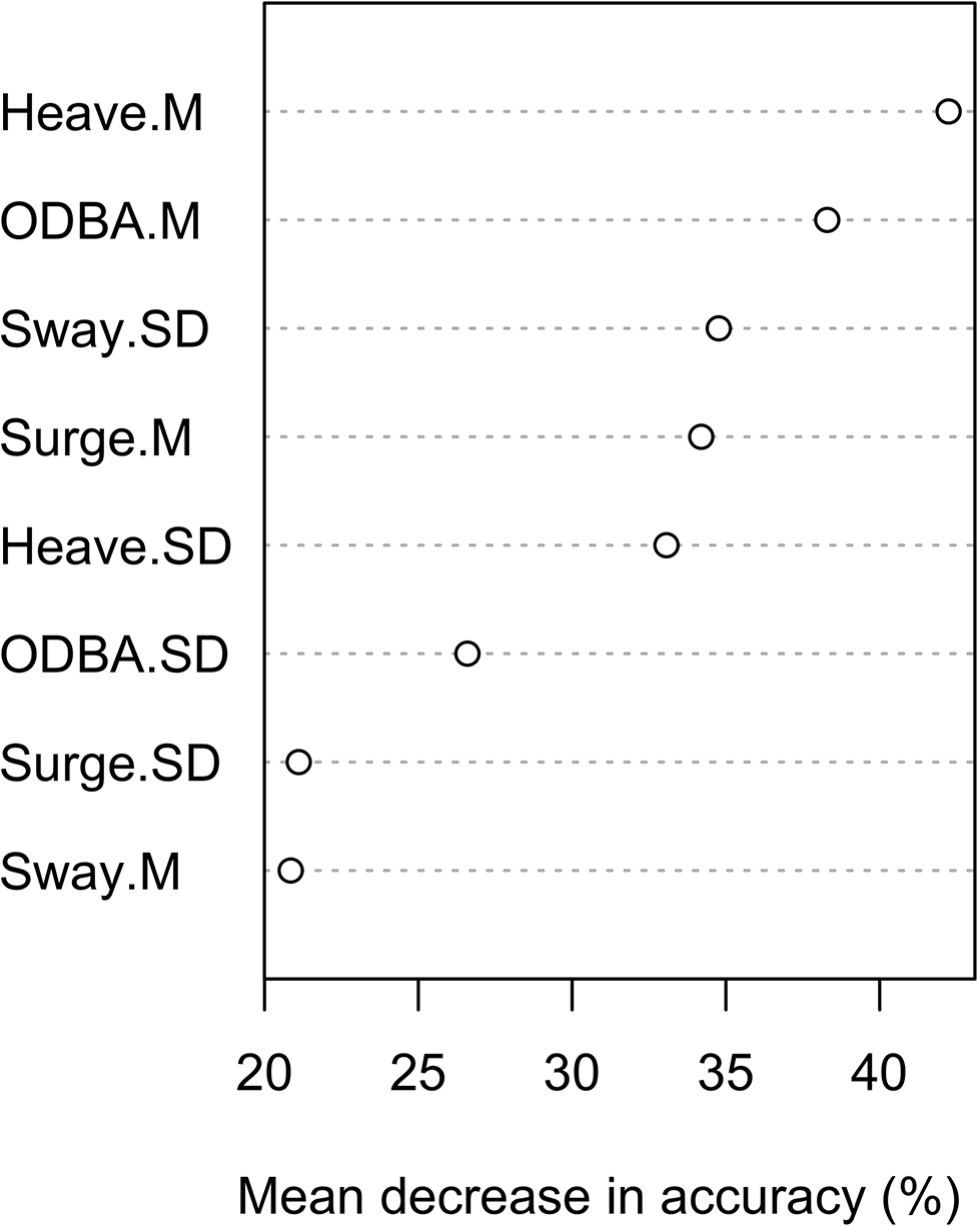
Variable importance plot for Eurasian beaver behaviours. A relative ranking of significant predictors, the permutation-based variable importance measure of the random forest model, is shown as the mean decrease in accuracy in percent. Higher values of mean decrease in accuracy indicate variables that contribute more to the accuracy of the classification.

In general, mean values were more important for classification than standard deviations, with the exception of the sway acceleration, where this relationship was inverted (Fig 3). As sway-heavy lateral movements (e.g. walking, swimming) are performed rhythmically, they result in a mean sway acceleration of about 0 g, a value that is common for all the behaviours observed in this study. Thus, the upper and lower peaks of the sway acceleration, as measured by the standard deviation, are more important for classification than the mean sway acceleration, which is least important (Fig 3). The mean heave acceleration proved to be most variable between behaviours and is therefore particularly important for classification (Fig 3).

**Fig 4:**
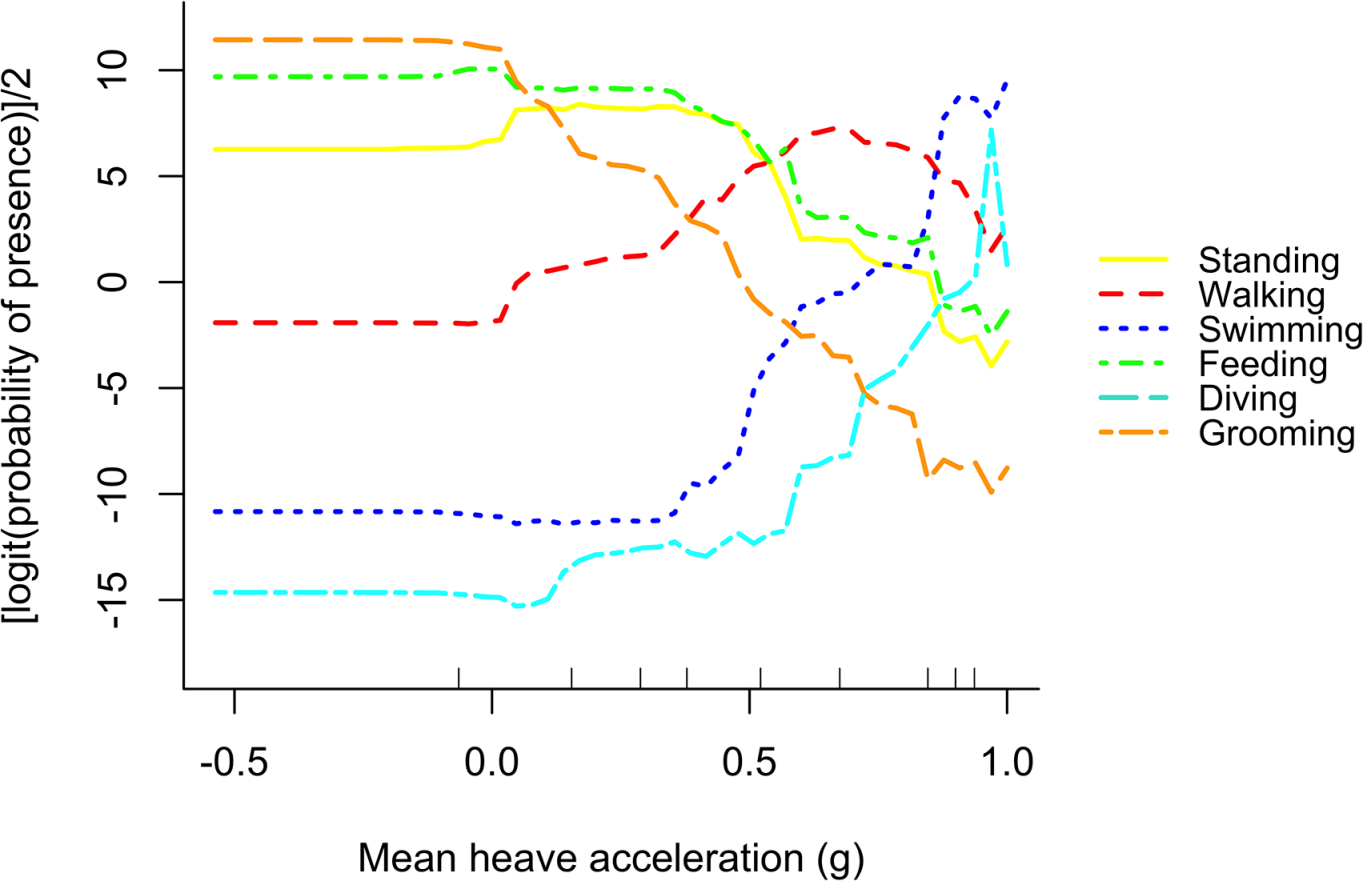
Partial dependence plot for the most important predictor variable, the mean heave acceleration. The partial dependence function plots a grid of values over the range of the mean heave acceleration on the x-axis, with decile rugs at the bottom of the plot representing the distribution of the total mean heave acceleration. The y-axis is on the logit scale and is centred to have zero mean over the data distribution.

A partial dependence plot of the mean heave acceleration shows the trend of this predictor after averaging out the effects of the other predictor variables in the model. The linear, horizontal trend at the beginning of each curve can be attributed to the sparseness of values in this range (see large decile). For grooming, feeding and standing, values between -0.5–0.5 *g* were good predictors, while values around 1 *g* indicate that it is unlikely the animal is performing one of these behaviours. Grooming shows the sharpest decline and thus mean heave acceleration values around 1 *g* are highly unlikely to be grooming. For walking, there was an upwards trend from 0 *g* with values between 0.6–0.9 *g* having the greatest predictive power. Swimming and diving show a sharp increase from 0.5 *g* on, indicating that values around 1 g are good predictors for these behaviours (Fig 4). In terms of postural changes, the upwards trend for walking, swimming and diving reflects the more bent-forward or stretched-out posture during these behaviours, while the downwards trend for standing, grooming and feeding indicates a more upright and leaned-back posture. In the following section, we refer primarily to mean heave acceleration values and illustrate the other axes when appropriate.

### Classification of behaviours

**Fig 5:**
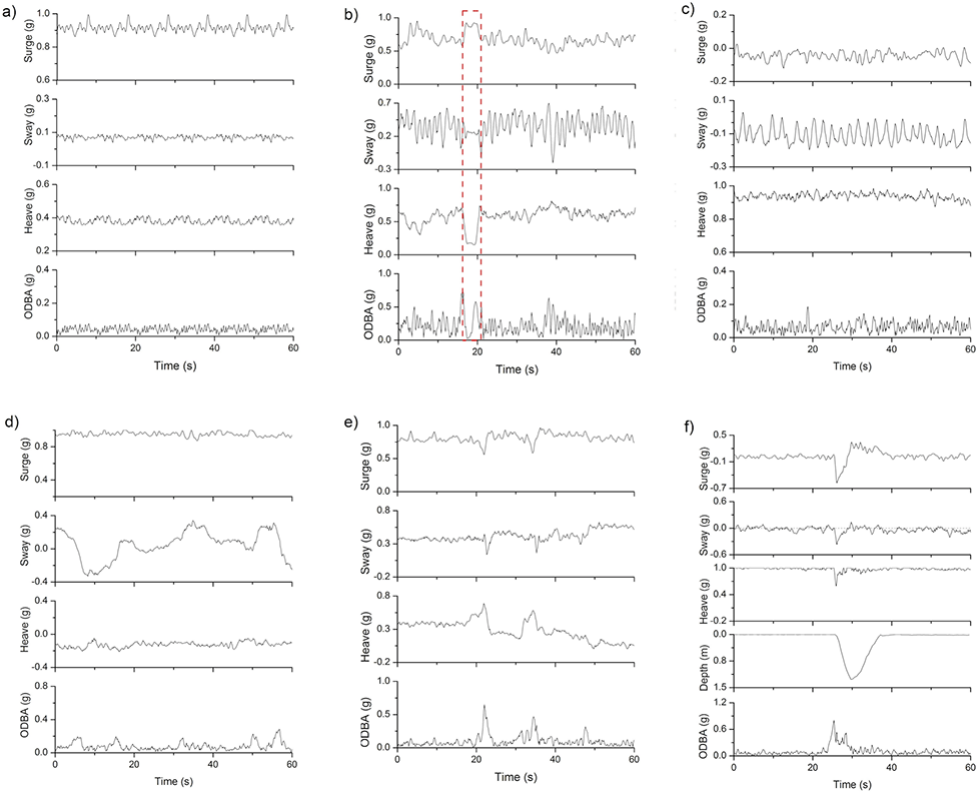
Changes in the acceleration signal during different Eurasian beaver behaviours. Examples of the static surge, sway and heave acceleration signals for Eurasian beavers during (a) standing, (b) walking (incl. a standing period within the dashed rectangle), (c) swimming, (d) grooming, (e) feeding and (f) a v-shaped dive with short bottom phase.

**Fig 6:**
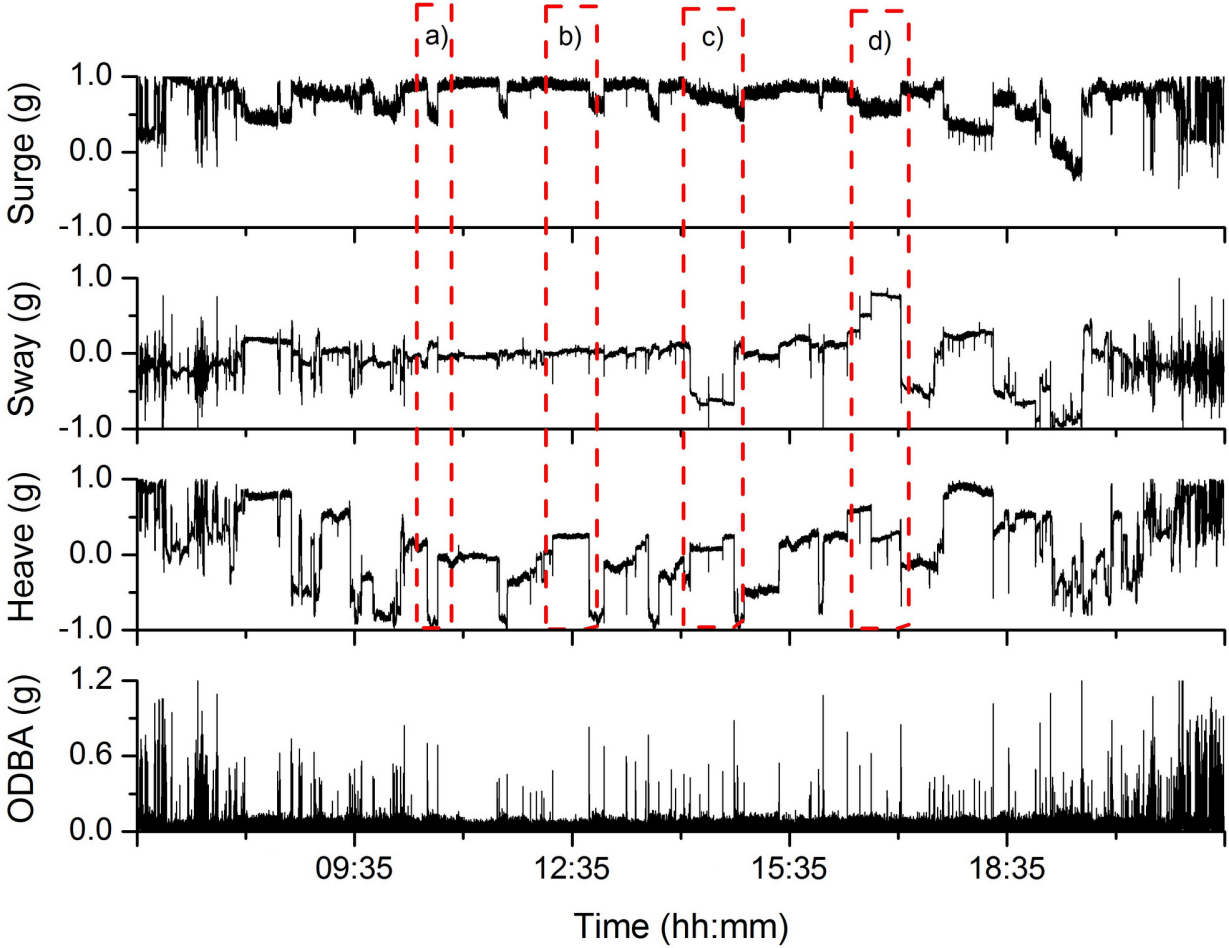
Changes in the acceleration signal of Eurasian beavers during a whole sleeping session. Example of the static surge, sway and heave acceleration of Eurasian beavers during a whole sleeping session. (a) Static heave acceleration values towards -1 *g* indicate lying on the back. (b) Static surge acceleration values towards +1 *g* and static sway acceleration values towards 0 *g* imply that the animal is sleeping in a sitting posture on its belly. (c) Static sway acceleration values towards -1 *g* indicate that the animal is lying on the left side, while (d) static sway acceleration values towards +1 *g* indicate lying on the right side.

Standing behaviour showed no appreciable temporal variation in any axis (Fig 5A, Table 2), while both walking and swimming were characterized by oscillations in the sway axis (Fig 5B and 5C, Table 2). Sway oscillations during walking showed greater amplitudes ($\bar{\text{x}}$ = 0.26 *g*, σ = 0.05 *g*) and lower cycle frequencies ($\bar{\text{x}}$ = 0.82 Hz, σ = 0.14 Hz) than during swimming (amplitude $\bar{\text{x}}$ = 0.11 *g*, σ = 0.07 *g*; cycle frequency $\bar{\text{x}}$ = 0.44 Hz, σ = 0.08 Hz). The wave pattern in the sway acceleration signal during walking originates from the beavers’ rolling gait, while oscillations in the sway acceleration during swimming represent propulsive, alternating leg strokes with the hind legs. Aside from having a negative mean heave static acceleration ($\bar{\text{x}}$ = -0.15 *g*, σ = 0.18 *g*), grooming also showed cyclic patterns in sway acceleration as the animals systematically undertook repetitive grooming motions, although amplitude over time was much more variable than in swimming or walking and the frequency much lower (Fig 5D, Table 2). Feeding was characterised by the lowest positive mean static heave acceleration ($\bar{\text{x}}$ = 0.19 *g*, σ = 0.13 *g*; Fig 5E). During diving, mean static heave acceleration was 0.91 *g* (σ = 0.08 *g*) and with different dive types being readily defined by data from the depth transducer (Fig 5F, Table 2). Sleeping behaviour was typified by very stable lying postures for long periods (normally minutes to hours) accompanied by occasional turns exemplified by radical changes in heave acceleration. Lying on the belly, on the sides, or on the back are illustrated in Fig 6. Gross mean ODBA values for various behaviours ranged from 0.056 *g* (σ = 0.013 *g*) for standing, to 0.233 *g* (σ = 0.046 *g*) and 0.265 *g* (σ = 0.029 *g*) during diving and walking, respectively (Table 2).

**Table 2:** Statistics of the static surge, sway and heave acceleration signal and overall dynamic body acceleration for the seven identified Eurasian beaver behaviours.

		Surge (*g*)		Sway (*g*)		Heave (*g*)		ODBA (*g*)			
Behaviour	n	$\bar{x}$	σ	$\bar{x}$	σ	$\bar{x}$	σ	$\bar{x}$	σ	Additional information	Fig
**Standing**	12	0.853	0.066	-0.004	0.097	0.360	0.136	0.056	0.013	No movement visible, thus the acceleration signal in all three axes is a straight line. Small variation in the heave acceleration channel due to different body angles, which are likely to represent different terrain slopes.	5A
**Walking**	12	0.624	0.131	-0.005	0.208	0.691	0.110	0.265	0.029	During walking, the beaver's posture is leaned forward ($\bar{\text{x}}$ surge and heave ≈ 0.6–0.7 *g*). The beaver's rolling gait is manifested as high, cyclic peaks in the sway acceleration (and therefore higher σ) where each peak represents a footstep.	5B
**Swimming**	12	0.128	0.104	-0.123	0.106	0.905	0.034	0.060	0.005	Due to the attachment on the lower back, swimming resulted in a mean heave acceleration close to 1 *g*, with a mean surge and sway acceleration around 0 *g*. Swimming beavers paddle with their hind legs, which results in low, cyclic peaks in the sway acceleration (see Fig 5C).	5C
**Grooming**	12	0.909	0.066	0.133	0.185	-0.149	0.185	0.075	0.020	When grooming, beavers usually sit on their hind legs and groom belly, and the left and right side of their back alternatively. This results in an undulated pattern in the sway acceleration channel (see Fig 5D), accompanied by an often negative heave acceleration due to leaned back posture.	5D
**Feeding**	12	0.919	0.048	-0.012	0.140	0.188	0.126	0.086	0.030	Feeding beavers adopt a more leaned back posture as in standing ($\bar{\text{x}}$ surge and heave ≈ 0.9 *g* and 0.2 *g*, resp.). The acceleration signal shows a steady sequence in all three channels, with betimes variation in the heave acceleration.	5E
**Diving** ^**a**^	8	0.100	0.210	0.120	0.100	0.910	0.080	0.233	0.046	A dive is initiated by a v-shaped peak in the heave and surge acceleration channel, but also clearly visible due to changes in the depth transducer (see Fig 5F).	5F
**Sleeping** ^**b**^	12	-	-	-	-	-	-	0.057	0.007	Sleeping consists of a series of steady sequences in all three channels, including occasional turning rates (see Fig 6).	6

^a^Similar-shaped, comparable dive types were only found in 8 individuals.

^b^Sleeping consists of a series of different postures (e.g. lying on the belly, on the sides, or on the back), thereby impeding the specification of mean values.

## Discussion

Accelerometry is a powerful tool for remotely studying animal ecology and by using this approach on beavers, we were able to categorize a set of seven different behaviours. Studies using accelerometers for identifying such a detailed suite of behaviours are rare, and this study is the one of the few to investigate a series of behaviours in a free-living mammal. A notable exception here is a study by Byrnes et al. [34], who were able to detect 5 locomotor behaviours in free-ranging Malayan colugos *Galeopterus variegatus*; including climbing, foraging, gliding, other behaviours and resting. Besides this, recent accelerometry studies have focused on wild felids, where researches used accelerometers integrated in GPS-collars to investigate behaviour and energetics of their study species. In cheetahs *Acinonyx jubatus*, Grünewälder et al. [32] were able to classify 5-min activity scores into a set of three key behaviours such as feeding, mobile and stationary. Similarly, Wang et al. [64] identified three behavioural categories in pumas *Puma concolor*, including low (walking) and high acceleration movement behaviours (trotting, running), and non-movement behaviours (resting, eating, grooming). On the same study species, Williams et al. [33] combined information from both GPS and acceleration sensors to investigate the energetics of instantaneous puma kills during three characteristic periods: pre-kill activity, pounce and kill and post-kill prey handling/eating. Apart from that, behavioural research using accelerometers on mammals has mainly focused on pets [23, 24, 65] or livestock [66, 67].

Research of this type on other animal classes includes a study by Tsuda et al. [20], who investigated the spawning behaviour of chum salmon *Oncorhynchus keta* and classified swimming, nosing, exploratory digging, nest digging, probing, oviposition, covering and post-spawning digging. Similarly, Whitney et al. [21] investigated mating behaviour of free-living nurse sharks *Ginglymostoma cirratum*, something that is, like the salmon study, facilitated by a sequence of highly conserved behaviours. Moreover, Lagarde et al. [45] used accelerometers on Greek tortoises *Testudo graeca* and distinguished five behavioural categories including immobility, feeding behaviours, digging, walking and sexual behaviours. Notwithstanding the pioneering work by Yoda et al. [5], Gómez Laich et al. [13] were the first to identify an independent series of behaviours in a free-living seabird species, the Imperial cormorant *Phalacrocorax atriceps*, where six behaviours including standing, sitting, floating on water, flying, walking and diving were identified. Together with the other studies cited above, we show the potential that accelerometers have for identifying the behaviour of free-living animals that cannot easily be observed (cf. [35]).

### Linking behaviour with acceleration signal

The static acceleration signals produced by the accelerometers are explicable in the light of the postural changes defining the different behaviours and the way this affects the orientation of the animal-attached logger. Firstly, the overall static values of the heave during standing, walking and swimming represent values that are, respectively, progressively angled to reflect the angle of the lower back. The beaver’s short-legged, plump body causes its rolling gait and sideways oscillations during walking; while during swimming leg-propulsion with the hind legs are responsible for sway oscillations. Grooming the belly and the back invariably involves the animal leaning back, which explains the mean negative static heave acceleration values. While using their forelegs to comb the water out of their pelage [18, 68], beavers typically start grooming their belly, then turn left and right, respectively, to groom their backs. These motions elicit the wave pattern in the sway acceleration, where individual differences in terms of time spent grooming each body part presumably cause the variation in the acceleration signal. Thus, the slightly positive mean sway acceleration indicates a higher grooming activity on the right side of the back which may reflect a tendency towards preferential use of the right paw, as found in other rodents such as mice [69] and rats [70, 71]. Feeding beavers adopt a seated posture while processing food items with their forelegs, often accompanied by occasional changes in position to facilitate another bite. These postural changes are apparent in the static heave acceleration. Finally, although diving behaviour can be identified definitively by changes in pressure, the systematic changes in body angle associated with the descent, bottom phase and ascent are definitive and are not characteristic of any other behaviour.

### Limitations in ability to deduce behaviour

The value of animal-attached accelerometers for determining behaviour depends on methodological issues and the hardware, or are related to the behaviour itself. Methodological issues are primarily related to the manner which the unit is mounted [72]. As pointed out by Gleiss et al. [73], accelerometers mounted on different sites on the body will give very different signals in response to both movement and posture, affecting the capacity of the researchers to determine behaviour. Similarly, the precise attachment mechanism should be identical between individuals, and preferably as stable as possible so that the unit records proper body movement rather than some concatenation of the body movement modulated by an unstable base. Practicalities may preclude the use of glue as used in this study, which creates a steady, stable support for the device. Despite the instability issue though, we note that collars have successfully been used for attaching accelerometers to animals [33, 65, 74].

Sampling frequency also plays a critical role in the ability of the tag to code for behaviours. Although the sampling frequency of 8 Hz allowed us to distinguish seven behaviours, any cyclical patterns in acceleration (analogous, for example, to the wave patterns produced in the sway acceleration during walking) will not be resolvable as such unless described by at least the Nyquist rate (at least twice of it’s naturally occurring frequency) [75]. Thus, a sampling frequency of 8 Hz will normally not allow resolution of any wave pattern with a frequency of greater than about 1.6 Hz, something that would, for example, make it impossible to determine the paw movements in grooming or the bites in gnawing. Similarly, highly transient behaviours may not be resolvable and simply contribute to the noise of the signal. We note that highly transient behaviours may not be resolvable even if the sampling frequency is high enough because not enough time is allocated to the activity for the pattern to be obvious. Thus, the binning duration is likely to play a role in determining the ability of dynamic body acceleration to help differentiate between transient behaviours with higher confidence with increasing time span over which the behaviour occurs.

Outside influences such as terrain, waves or wind can alter posture and induce variation in the manifestation of a behaviour via accelerometry. In our study area, there was great diversity in river bank types, ranging from steep, rocky slopes to grassy, flat banks. The consequence of this was that walking, in particular, sometimes involved heave acceleration values at considerable variance than would normally be exhibited, albeit for short periods. Even though waves can influence posture in water, this seemed to occur little in our study. Similarly, wind speed may alter flying behaviour in birds and should be taken into account where relevant [41, 76].

In addition, there are two other main issues that limit the use of accelerometry to identify animal behaviour. The first one relates to animals performing multiple behaviours at the same time, such as walking and eating. In this case, it is likely that behaviour with the higher amplitude overshadows the other behaviour and is dominant in the acceleration signal. The second issue is related to behaviours exhibiting little movement such as resting and sleeping. In this study, we were able to differentiate those two behaviours by picking resting periods when the animal was outside the lodge. Still, both cases may hamper the correct assignment of acceleration data to behavioural classes.

## Conclusions and Perspectives

Our study demonstrates that acceleration data loggers can be used to elucidate the behaviour of free-living animals with only minor disturbance. Model outputs, such as the random forests classification applied in this study, can be used to inform computer programs to search automatically for behaviours, although it is unclear how well such systems will deal with problems such as transience between behaviours. There are obvious current limitations on software, particularly software that has the flexibility to work for multiple species with differing acceleration signatures. Ultimately, it may be possible to treat animals according to type, such as tetrapods versus birds, but this will require an extensive multi-species database to inform development. Other avenues such as spectral analysis [77] and machine learning approaches (cf. [78]) will also be useful as well as powerful visualization techniques such as spherical plots [79]. Developments in the solid state industry fuelled by, for example, mobile phones, will inevitably lead to the development of smaller acceleration units with increased battery longevity and storage capacity that operate with higher sampling frequencies. The new generation of tags, coupled with appropriate software, will lead to systems that can be deployed for months or years and allow identification of highly complex behavioural traits such as social interactions or behaviours with highly varying appearance. Ultimately, it seems likely that this approach will be implemented on pest [80] and endangered wildlife [81, 82] alike to help inform management and conservation actions [83, 84].

## Supporting Information

S1 File Dataset. **Static acceleration values (*g*) and overall dynamic body acceleration (ODBA) (*g*) during six Eurasian beaver behaviours.** Means and standard deviations of the static surge, sway and heave acceleration signal and ODBA for beavers during standing, walking, swimming, grooming, feeding and diving.
(CSV)

S2 File Ethics statement documents. (ZIP)
