# Supplementary material for: The Use of Acceleration to Code for Animal Behaviours; A Case Study in Free-Ranging Eurasian Beavers Castor fiber
Source: PLoS One. 2015 Aug 28;10(8):e0136751. doi: 10.1371/journal.pone.0136751 (PMC4552556; doi:10.1371/journal.pone.0136751)
Supplement: S2 File — (ZIP) [file pone.0136751.s002.zip › Ethics statements docs/Direktoratet for naturforvaltning 09-11.pdf]

Høgskolen i Telemark

3800 Bø I Telemark
Att. Frank Rosell

Deres ref.:	Vår ref. (bes oppgitt ved svar): **2008/14367 ART-VI-ID** Arkivkode: **444.5**	Dato: **06.01.2009**

Tillatelse til fangst og merking av bever i årene 2009-2011

Vi viser til søknad av 11.oktober 2008 om tillatelse til levendefangst og merking av bever.

Høgskolen i Telemark ved prosjektleder Frank Rosell gis med dette tillatelse til innfanging og merking av inntil 100 bever per år, i årene 2009, 2010 og 2011 i kommunene Bø, Sauherad og Nome i Telemark i henhold til søknad.

Tillatelsen gis med hjemmel i forskrift om innfanging og innsamling av vilt for vitenskapelige eller andre særlige formål, fastsatt av Direktoratet for naturforvaltning den 14. mars 2003, jf. Lov om viltet § 26 nr. 4.

Prosjektleder kan bemyndige andre til å foreta innfangingen. Skriftlig bemyndigelse med henvisning til denne tillatelsen skal medbringes under innfangingen.

Direktoratet setter følgende generelle vilkår for tillatelsen:

- Tillatelsen gjelder i 2009, 2010 og 2011.
- Grunneiers tillatelse må innhentes og kommune samt lokal politimyndighet må varsles før fangst tar til.
- Fangst/immobilisering av dyr skal skje i samsvar med forsøksdyrutvalgets tillatelse.
- Rapport fra perioden oversendes DN innen slutten av 2011.
- Denne tillatelse skal alltid medbringes under fangst/merking.
- Vedtaket kan påklages til Miljøverndepartementet innen tre uker. Eventuell klage sendes via DN.

Med hilsen

Terje Bø
Terje Bø e.f.
Seksjonssjef

Reinkind
Ingrid Regina Reinkind
