# Supplementary material for: The Use of Acceleration to Code for Animal Behaviours; A Case Study in Free-Ranging Eurasian Beavers Castor fiber
Source: PLoS One. 2015 Aug 28;10(8):e0136751. doi: 10.1371/journal.pone.0136751 (PMC4552556; doi:10.1371/journal.pone.0136751)
Supplement: S2 File — (ZIP) [file pone.0136751.s002.zip › Ethics statements docs/Forsoksdyrutvalget 2008.pdf]

FORSØKSDYRUTVALGET

Deres ref:

Vår ref:
S-2007/101839

Dato:
18. februar 2008

Høgskolen i Telemark v/Frank Rosell
3800 Bø i Telemark

ANG. SØKNAD OM FORSØK MED BEVER
Sak behandlet i forsøksdyrutvalgets møte 29/1-08.

Dokumenter i saken:
5.6.1 PDF av søknad i FOTS (id 742) fra Frank Rosell, datert 11/12-07.

Utvalgets behandling:
Vedtak:
Utvalget godkjenner forsøket iht. søknaden, men det presiseres at godkjenning bare kan gis for 2 år, jf forskrift 15 jan 1996 nr. 23 om forsøk med dyr §§ 7, 8 og 10.

Begrunnelse:
Søknad om forsøk er sendt inn innen den fastsatte fristen og inneholder tilstrekkelige opplysninger til at utvalget kan fatte vedtak.

Utvalget anser også det såkalte Y-rør forsøket å være en del av det godkjenningspliktige forsøket. Utvalget ser all innfanging av ville dyr til forsøk, som en del av det som krever godkjenning.

Hensikten med forsøket, å benytte 100 bever for å studere spredningsstrategier, og den planlagte gjennomføringen er av vitenskapelig og samfunnsmessig betydning slik at de generelle vilkår i forsøksdyrforskriftens § 8, første ledd er oppfylt. Det foreligger ikke anvendelige alternativer til bruk av levende dyr som beskrevet i § 8, tredje ledd. For forsøk utover 2 år må det sendes ny søknad, jf. forsøksdyrforskriftens § 10, 3. ledd.

Det forutsettes at søker oppfyller de generelle vilkår for forsøk i forsøksdyrforskriften. Utvalget finner det ikke nødvendig å sette spesielle vilkår for dette forsøket utenom det som allerede er angitt i søknaden.

Eventuelle avvik og endringer fra den godkjente søknaden må meddeles skriftlig til utvalget og evt. som søknad om endring av forsøket.

Utvalget minner om at institusjon, bedrift eller person som har fått tillatelse til å utføre forsøk med levende dyr skal rapportere om antall benyttede dyr i det aktuelle kalenderår, jf forsøksdyrforskriften § 24.

Vedtak kan påklages til Mattilsynet, jfr. lov 10 feb 1967 om behandlingsmåten i forvaltningssaker (forvaltningsloven) § 28. Klagefristen er 3 uker fra mottak av dette brev, jfr. forvaltningsloven § 29. Klagen stiles til Mattilsynet, Hovedkontoret, men sendes via Forsøksdyrutvalget.

Med hilsen for Forsøksdyrutvalget

Bjørn Groven

Bjørn Groven
Sekretær

Adresse: Forsøksdyrutvalget
c/o Mattilsynet, Felles postmottak
Postboks 383
N- 2381 Brumunddal

Telefaks: 23 21 68 01
e-post: postmottak@mattilsynet.no
Hjemmeside: www.fdu.no

Sekretariat: Bjørn Groven 23 21 66 48
Gunvor Knudsen 23 21 66 63
Jorunn Sørensen 23 21 65 24
