# Supplementary material for: The Use of Acceleration to Code for Animal Behaviours; A Case Study in Free-Ranging Eurasian Beavers Castor fiber
Source: PLoS One. 2015 Aug 28;10(8):e0136751. doi: 10.1371/journal.pone.0136751 (PMC4552556; doi:10.1371/journal.pone.0136751)
Supplement: S2 File — (ZIP) [file pone.0136751.s002.zip › Ethics statements docs/Forsokydyrutvalget 2009.pdf]

FORSØKSDYRUTVALGET

Deres ref: | Vår ref: 2009/137495 | Dato: 23.11.2009

Høgskolen i Telemark
Postboks 203
3901 Porsgrunn

Att. Frank Rosell

VEDTAK I FORSØKSDYRUTVALGET - FOTS ID 2170

Sak 5.3 behandlet i Forsøksdyrutvalgets møte 17.11.2009.

Dokumenter i saken:
5.3.1 Pdf av søknad i FOTS (id 2170) fra Frank Rosell, datert 23/10-09.
5.3.2 Vedlegg til søknaden (3 stk), det ene vedlegget er en artikkel på 28 sider, den legges frem i møtet.

Vedtak:
Utvalget godkjenner forsøket, iht. søknaden, men fram til 31/12-12, jf forskrift 15 jan 1996 nr. 23 om forsøk med dyr §§ 7, 8 og 10. Utvalget forutsetter at alle som deltar i forsøket er angitt i søknaden og at de har opplæring i forsøksdyrlære tilpasset den funksjon de har i forsøket jf. forsøksdyrforskriften § 12, tredje ledd og § 13, første ledd.

Begrunnelse:
Søknad om forsøk er sendt inn innen den fastsatte fristen og inneholder tilstrekkelige opplysninger til at utvalget kan fatte vedtak.

Utvalget finner at hensikten med forsøket, å registrere aktivitetsmønsteret hos bever, og den planlagte gjennomføringen, å merke (med GPS-sendere, dykker-loggere eller VHF-sendere) og ta prøver av 100 bever, er av vitenskapelig og samfunnsmessig betydning slik at de generelle vilkår i forsøksdyrforskriftens § 8, første ledd er oppfylt. Det foreligger ikke anvendelige alternativer til bruk av levende dyr som beskrevet i § 8, tredje ledd

Godkjenningsperioden er satt til 2 år, jf. forskrift 15 jan 1996 nr. 23 om forsøk med dyr §§ 10, 3. ledd.

Eventuelle avvik og endringer fra den godkjente søknaden må meddeles skriftlig til utvalget og evt. som søknad om endring av forsøket.

Vedtak kan påklages til Mattilsynet, jf. lov 10. februar 1967 om behandlingsmåten i forvaltningssaker (forvaltningsloven) § 28. Klagefristen er 3 uker fra mottak av dette brev, jf. forvaltningsloven § 29. Klagen stiles til Mattilsynet, Hovedkontoret, men sendes via Forsøksdyrutvalget.

Med hilsen
for Forsøksdyrutvalget

Gunvor Knudsen

Gunvor Kristin Knudsen
FDUs sekretariat

Adresse:
Forsøksdyrutvalget
c/o Mattilsynet, Felles postmottak
Postboks 383
N- 2381 Brumunddal

Telefaks: **23 21 68 01**
e-post: postmottak@mattilsynet.no
Hjemmeside: www.fdu.no

Sekretariat:
Gunvor Knudsen **23 21 66 63**
Johanne Holmen **23 21 66 92**
